# An accelerating inverted wing with ground effect: downforce measurement and reconstruction

**DOI:** 10.1007/s00348-026-04191-5

**Published:** 2026-05-25

**Authors:** Shanwei Zhou, Edwin F. J. Overmars, Jerry Westerweel

**Affiliations:** Laboratory for Aero & Hydrodynamics, Delft University of Technology, Mekelweg 2, 2628 CD Delft, The Netherlands

**Keywords:** Ground effect, Flow separation, Impulsive flow, Particle image velocimetry, Formula 1 racing

## Abstract

This paper presents an experiment designed to measure the downforce generated by an inverted wing under ground effect conditions, during both accelerating and steady motion. The flow around the wing is captured using high-speed planar particle image velocimetry, allowing for detailed observation of the flow field throughout the motion. A multi-body impulse-based method is employed to reconstruct the downforce from the measured flow field. To further explore how flow structures contribute to downforce generation, lift coefficient contours are analyzed. The results show that lower ground clearance produces greater downforce during acceleration, primarily due to the boundary layer on the suction side of the wing. Following acceleration, flow separation occurs, and the wake region begins to contribute significantly to downforce. Based on these findings, recommendations for future aerodynamic designs are proposed that may be relevant in Formula 1 racing.

**Supplementary Information:**

The online version contains supplementary material available at 10.1007/s00348-026-04191-5.

## Introduction

The start of a Formula 1 (F1) race is considered a critical moment of a race, characterized by the ‘launch’ (Preosti 2021). This moment often features significant position changes that can shape the outcome of the race (Gasparetto et al. 2024). During the start, an F1 car accelerates from standstill in a straight line toward the first corner, with a typical acceleration of 18 m/s$^2$. The amount of power that can be effectively used during this acceleration is limited by the grip of the tires (Katz 2006; Toet 2013). The *International Automobile Federation* (FIA) stipulated regulations that restrict variations in the design of many aspects of the race car, with the exception of aerodynamic components. These aerodynamic components are key, as they generate the so-called downforce, increasing tire grip and improving acceleration performance (Katz 2006).

The primary objective of aerodynamic development in Formula 1 is to maximize the downforce while minimizing the associated increase in drag (Katz 2006). Among the various aerodynamic components of an F1 car, the front wing operates under relatively uniform inflow conditions (Toet 2013) and contributes substantially to the overall downforce generation (Knapik et al. 2018). In this study, we investigate the downforce of the front wing of the Tyrrell 026 F1 race car, which is a modified NASA GA(W) LS(1)-0413 wing, during acceleration. The wing profile coordinates are provided by Zerihan (2001). This wing profile is widely used in experimental and numerical research (Zerihan 2001; Arrondeau and Rana 2020; He et al. 2014), and has since become a standard validation case for inverted wings in ground effect (Doig and Barber 2011).

The front wing is typically mounted very close to the ground. (Zerihan (2001)) demonstrated that the ground effect can significantly influence the downforce under steady flow conditions; see also Pistolesi (1937), Zhang et al. (2006), Cui and Zhang (2010) and Lee and Lin (2022). Unsteady cases are studied as well, often in relation to biological propulsion (e.g., Quinn et al. 2014). However, for an accelerating wing with fixed angle of attack (AoA), the impact of the ground effect remains largely unexplored. This led to our current investigation.

**Fig. 1:**
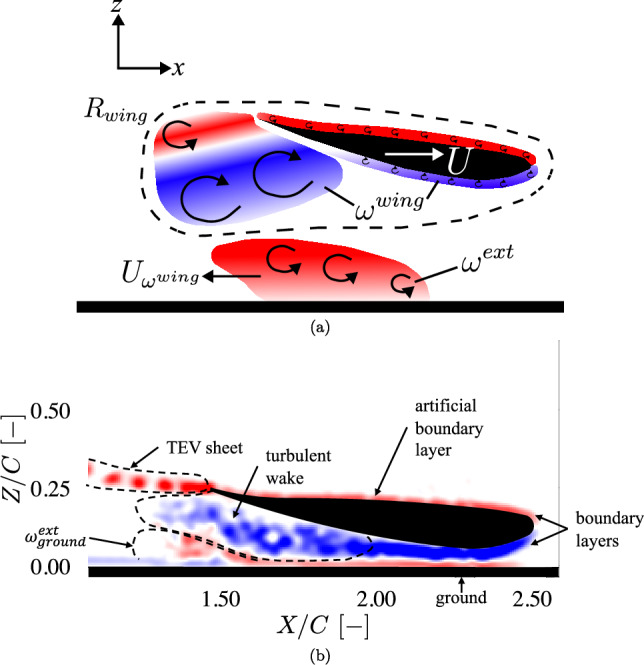
**a** Schematic representation of the typical flow field of the inverted wing accelerating with ground effect, following Gehlert et al. (2023). The region $R_\textrm{wing}$, enclosed by the dashed line, contains the wing-generated vorticity $\omega ^\textrm{wing}$. The external vorticity $\omega ^\textrm{ext}$ is advected at a velocity $U_{\omega ^\textrm{wing}}$ induced by the wing-generated vorticity following the Biot-Savart law; see subsubsect. 3.2.1. **b** Typical example of a measured flow field. The colors represent positive (red) and negative (blue) values of the out-of-plane component of the vorticity. The black dashed lines define different flow structures, like the wake region, the trailing edge vortex sheet (TEV sheet), the wing boundary layers, and the vorticity generated near the ground $\omega ^\textrm{ext}_\textrm{ground}$. The “artificial boundary layer” on the pressure side of the wing is interpolated from the measurement data in the region obscured by the wing; see Fig. 5

We use an experimental setup (further described in Sect. 2) to investigate the ground effect of the Tyrrell wing, where we consider different ground clearances with a fixed angle of attack (AoA) of $\alpha = 6.60$°. This AoA is chosen based on the previous research by Zerihan (2001), who varied the AoA between $\alpha = -3$°and $\alpha$ = 9°. We measure the flow field around the wing using planar (2D) particle image velocimetry (PIV). A typical result of the measured flow field is presented in Fig. 1, which shows the out-of-plane component of the vorticity that is generated jointly by the wing and the ground. The vorticity generated by the wing includes a trailing edge vortex (TEV) sheet, a wake region, and a boundary layer surrounding the wing. We use the multi-body impulse-based method introduced by Gehlert et al. (2023) to link the measured flow field to the measured downforce.

The objective of this research is to improve the understanding of the aerodynamic performance of a wing accelerating with ground effect. Based on the results, we aim to provide recommendations for optimizing ground clearance during launch, as well as guidance for future research on unsteady flow and ground effect interactions.

An outline of this paper is as follows. In sect. 2 we describe the experimental setup, including the wing kinematics, force measurement, and PIV measurements. The results are described in subsect. 3.1, and in subsect. 3.2 we describe what aspects of the flow field contribute to the downforce. The conclusions of this study are summarized in sect. 4.

## Experimental set-up

The experimental facility consists of a gantry robot (Reis Robotics RL50) that translates a 3D-printed wing through a water-filled tank. The downforce is measured using a force/torque transducer (Delta IP60, ATI, USA) attached to the robot. The flow around the wing is measured by planar high-speed particle image velocimetry (PIV). The robot is programmed to accelerate the wing at a constant rate from rest to a predetermined constant velocity, ensuring a controlled motion throughout the experiment. This precise movement allows for consistent and repeatable measurements. This flow facility is identical to the one used by Grift et al. (2019), to which we refer for further details.

**Fig. 2:**
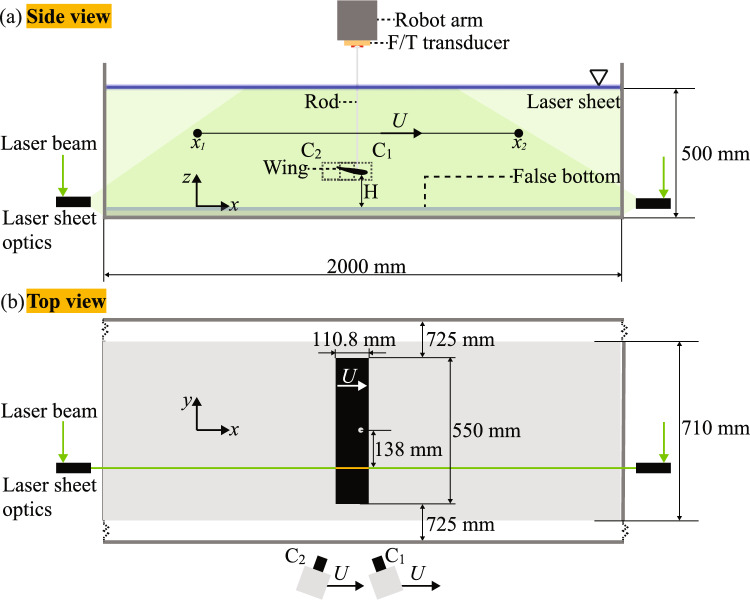
Schematic of the experimental facility, which is almost identical to that of Grift et al. (2019). The setup consists of a 2.0$\times 2.0 \times 0.5~\hbox {m}^3$ water-filled glass tank with a 2.00$\times$0.71 m$^2$ transparent false bottom. The *x*-axis is in the direction of motion, with the origin at the start position in the middle of the tank side walls and at the top surface of the false bottom (in gray); the *y*-axis is in the transverse direction, and *z*-axis upward and normal to the surface of the false bottom. **a** Side view of the set-up. The wing travels along a straight line, from $x_1$ to $x_2$, at a constant ground clearance *H*. The ground clearance *H* is the minimum distance between the wing and the false bottom. The wing travels with a velocity *U*(*t*), shown in Fig. 4. The blue line indicates the free water surface. The laser beam is split and illuminates the wing from opposite sides to avoid shadows (green shaded areas). The wing (black) is drawn at the same scale as the tank. **b** Top view of the set-up. The flow around the wing and over the false bottom is recorded by two cameras, $\textrm{C1}$ and $\textrm{C2}$, that move along with the wing. The wing is placed at the middle of the tank. The laser light sheet (green) is positioned at a quarter of the spanwise width from the wing center; this is halfway between the rod and the end plate

One of the key variables in this study is the ground clearance *H*, which is represented by the distance between the wing and the false bottom; see Fig. 2. By varying this distance, we study the ground effect on the downforce of the wing.

**Fig. 3:**
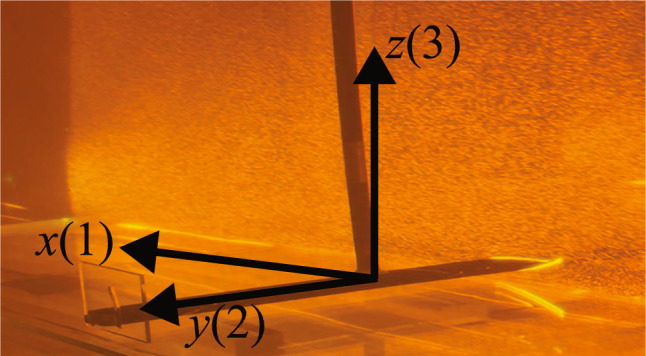
Definition of the Lagrangian coordinate system of the wing, indicating the three directions for the measured forces (black arrows). The numbers refer to the coordinates for the added mass tensor in Eq. 2. The background photo is taken with an orange filter during one of the PIV measurements

**Fig. 4:**
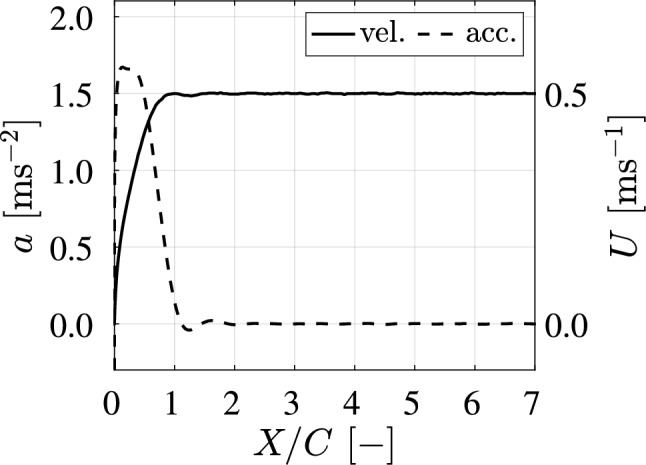
The acceleration *a* and velocity *U* of the wing as a function of the traveled distance *X* relative to the wing chord length *C*. From $X/C = 0$ to $X/C = 1$, the wing accelerates from zero velocity to $U = 0.5~ \hbox {m}\,\hbox {s}^{-1}$, with acceleration $a\,=\,1.64~\hbox {m}\,\hbox {s}^{-2}$. For $X/C> 1$, the robot applies a constant velocity

**Fig. 5:**
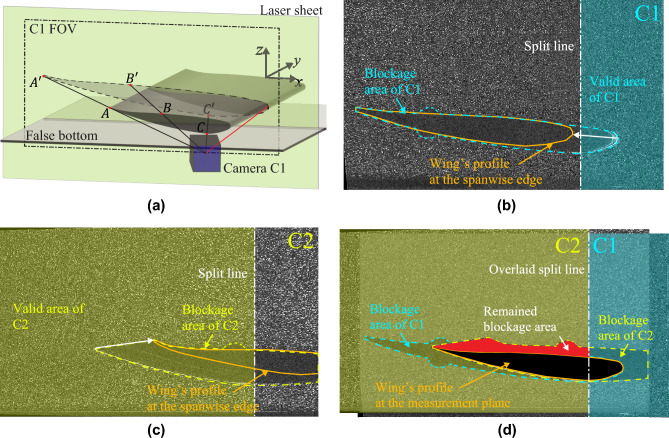
The projection effect of the tip of the wing closest to the camera onto the measurement plane. Because of this effect, a part of the images is obscured. The obscured area is different for the two cameras $\textrm{C1}$ and $\textrm{C2}$ (as shown in Fig. 2). To maximize the view on the suction side, both cameras are angled to look underneath the wing. **a** A schematic of the projection effect, using the field of view (FOV) of $\textrm{C1}$ as an example. The obscured area on the measurement plane is marked out by dashed line and filled with transparent gray. **b** & **c** Typical raw images recorded by $\textrm{C1}$ and $\textrm{C2}$, theme colored in blue and yellow, respectively. The obscured areas are marked out by dashed lines. **d** Combining the FOVs of $\textrm{C1}$ and $\textrm{C2}$ to achieve the least obscured total view. The area taken from $\textrm{C1}$ is colored in blue; the area taken from $\textrm{C2}$ is colored in yellow. The remained blockage area, which is near the pressure side of the wing, is colored in red. The flow field in this area is interpolated following the procedure introduced by Gehlert et al. (2023)

### Wing model

The wing used in the experiment has a two-dimensional profile with a span $B=550\,{\textrm{mm}}$ and a chord $C=110.8\,{\textrm{mm}}$. End plates with a length of 120 mm and a height of 60 mm are applied to both spanwise edges of the wing, in order to closely approximate a two-dimensional flow around the wing. The wing is 3D-printed with acrylonitrile butadiene styrene (ABS), and the end plates are made of acrylic. A photo of the wing taken during one of the PIV measurements is shown in Fig. 3. A Lagrangian coordinate system is defined with its origin at the geometric center of the wing.

The wing is coated with a black light-absorbing paint.^1^ This paint has a nominal absorption coefficient of 99.4%, and thus significantly reduces effects due to reflections and glare from the illuminating laser light sheet that may interfere with the PIV measurements.

To detect the edges of the wing under laser illumination, fluorescent orange paint is applied at the position where the laser light sheet hits the wing surface. The fluorescent paint absorbs green laser light and re-emits it as orange light, resulting in a clearly visible line in the recorded images that defines the wing surface profile during the PIV measurements.

### Kinematics

Fig. 2 shows the robot arm holding the wing. The gantry robot moves the wing from its start position $x_1$ to its end position $x_2$. The distance traveled by the wing is indicated as,

1 $$\begin{aligned} X(t) = x(t)-x_1. \end{aligned}$$

The tank has a bottom surface area of 2.00$\times$2.00 m$^2$ with 0.60 m high side walls, and is filled with water to a height of 0.50 m. A false bottom is introduced to provide a horizontal plane surface; see Fig. 2.

The wing is accelerated from standstill to a constant velocity along a straight line, covering a total distance of 1.15 m. The distance from the wing to the vertical walls of the tank is at least six times the chord length in any horizontal direction. The clearance between the bottom of the wing and the false bottom is varied between 6 and 155 mm.

The gantry robot records the wing position at a rate of 92 Hz and has a repeatability of the position better than 0.100 mm with a resolution of 1 $\mu$m (Grift et al. 2019). The recording results are presented in an Eulerian coordinate system with the *x*-axis in the direction of the wing motion, the *y*-axis in the spanwise direction of the wing, and the *z*-axis normal to the surface of the false bottom pointing upwards. The origin of the Eulerian coordinate system is at the center of the wing at the starting position $x_1$ of the wing; see Fig. 2.

The wing is accelerated along a straight path to a velocity of $U = 0.50~\hbox {m}\,\hbox {s}^{-2}$ with an acceleration of $a\,=\,1.64~\hbox {m}\,\hbox {s}^{-2}$; see Fig. 4. The time to reach the target velocity is 0.45 s; the corresponding distance *X* traveled by the wing is one chord length, i.e., *X*/*C* = 1.0. This acceleration is the highest achievable acceleration for the gantry robot.

At *U* = 0.5 ms$^{-1}$ the Reynolds number is *Re* = 5.5$\times$10$^4$, given by the chord length *C*, and the kinematic viscosity $\nu (= 1.0\times 10^{-6}\,\hbox {m}^{2}\,\hbox {s}^{-1}$). This is the highest achievable Reynolds number given the dimensions of the tank. This kinematic setup would represent a front wing of an F1 car that accelerates from standstill to $11\,\mathrm{km/h}$. Since an F1 car can reach $300\,\mathrm{km/h}$ during the launch, the typical Reynolds number of a front wing becomes $\mathcal {O}(10^6)$. While the Reynolds number in this experiment is smaller than the real situation, this experiment is a step toward more realistic conditions where acceleration was previously ignored. Future research with a wing accelerating to such a Reynolds number is still required.

### Force measurement

Figure 3 defines the Lagrangian coordinate system used in the measurement of the forces considered in this study. The forces on the wing are measured with a force/torque transducer (Delta IP60, ATI, USA). The transducer is mounted between the robot arm and the cylindrical rod that holds the wing, as shown in Figs. 2 and 3. The force data are acquired at a rate of 10 kHz. The downforce is measured as the *z*-component of the force. Any contribution of the flow around the rod to the measurement of the downforce is considered negligible, as verified by performing measurements with the rod in place but without the wing attached to it. The Lagrangian coordinate system is also used for computing the added mass; see subsubsect. 3.1.1.

### Particle image velocimetry

Simultaneously with the force measurements, planar high-speed particle image velocimetry (PIV) is used to visualize and measure the flow field around the wing. The system uses two cameras that are mounted on the robot to move along with the wing; this is further explained below. The flow field is illuminated with a thin planar light sheet parallel to the *x*-*z* plane that crosses the wing at a distance of 138 mm from the center of the wing; this is halfway the rod that supports the wing and the end plate at the end of the wing; see Fig. 2. We thus avoid significant flow disturbances from either the rod or the end plate, with the aim to approximate a two-dimensional flow field in the measurement plane. The laser light sheet is generated from the beam of a high-speed Nd:YLF laser (Litron LDY304-PIV). The laser delivers an energy of 40 mJ per pulse. Using a combination of beam splitters and lenses, the laser beam is split into two beams that each create a thin light sheet from opposite sides of the wing toward the leading and trailing edges of the wing along the *x*-axis; see Fig. 2. This configuration avoids shadows where no particle images can be recorded.

At the back of the wing, a plano-concave cylindrical lens with a focal length of 12.5 mm is used to convert the beam into a sheet; a spherical plano-convex lens of with a 700 mm focal length is used to focus the light into a thin sheet. At the front of the wing, the optics consists of a combination of a plano-concave cylindrical lens with a focal length of 25 mm and a 2000-mm focal length spherical plano-convex lens. The thickness of the combined laser sheet is less than 1.5 mm in the measurement domain.

The water-filled tank is seeded with small fluorescent tracer particles (Cospheric) with a nominal density of 995 kg m$^{-3}$. The tracer particles have diameters between 53 and 63 $\mu$m. The peak emission of the fluorescent light is 607 nm. To filter out the green laser light (527 nm wavelength) both cameras are equipped with optical long-pass filters (Schott OG590).

Two high-speed cameras (Phantom VEO 640 L) record the motion of the tracer particles in the light sheet. Each camera has an image format of 2560$\times$1600 pixels.

The cameras are equipped with 55-mm focal length lenses (Nikon AF Micro Nikkor 55 mm f/2.8). An aperture number of $f^{\#}$ = 5.6 gives a depth of field of 4.0 mm that includes the light sheet thickness, so that small and focused particle images are recorded. Scheimpflug adapters are placed between the lens and the camera body to tilt the image sensor so that the plane of focus aligns with the image plane. The cameras have a field of view of 180.0$\times$112.5 mm$^2$, which gives a scaling factor of 0.07031 mm/pixel. The high-speed cameras and the laser are synchronized to record images at 1278 frames per second (fps). Each run consists of a total of 3058 images.

As illustrated in Fig. 5, the spanwise tip of the wing closest to either of the cameras obscures part of the field of view of each camera. Using two cameras, where one camera (C1) has an unobstructed view of the leading edge of the wing and the other camera (C2) an unobstructed view of the trailing edge of the wing, the combined view captures the most significant part of the flow around the wing. The cameras observe the flow slightly from below the wing; therefore, only a small section of the flow above the wing is not measured. This is the pressure side of the wing, where the flow is considered irrotational with a thin boundary layer on the top surface of the wing, which is considered to have a small and negligible contribution to the total downforce; this is further discussed in subsubsect. 3.2.3. Following Corkery et al. (2019) and Gehlert et al. (2023), the absent flow data in the commonly obscured area are interpolated from the surrounding measured flow field and applying the no-slip boundary condition at the wing surface.

We use commercial software (DaVis 10.2, LaVision GmbH, Germany) for the acquisition of the images. The image mapping function is determined using a calibration target. The calibration procedure first determines the image mapping function. The image is then de-warped to remove perspective distortion, after which the known geometry of the calibration target yields the physical scaling from pixels to real-world units. The images are then processed using in-house developed Matlab functions that perform the PIV analysis as described by Adrian and Westerweel (2011). Subsequent image pairs are interrogated using a multi-grid PIV algorithm with an interrogation window size of 48$\times$48 pixels for the first pass and 24$\times$24 pixels for the second pass. A 50% overlap between adjacent interrogation positions is used, resulting in measured velocity fields with a data spacing of 0.84 mm. Data validation and post-processing are applied, to reach 98% valid data in the area of interest, specifically in the wake region of the wing. Detected outliers are replaced by interpolating surrounding valid data.

## Results

### Ground effect in accelerated motion

To better understand the ground effect, both force and PIV measurements are conducted. In particular, for accelerated motion in a dense fluid, like water, one should also include the added mass force to describe the forces. In this section, we first discuss the added mass contribution, which is followed by interpretation of the downforce in terms of a lift coefficient. We then examine the measured vorticity field at two representative ground clearances. The relation between the vorticity field and the downforce will be discussed in subsection 3.2, using the impulse-based method introduced by Gehlert et al. (2023).

#### Added mass

Corkery et al. (2019) and Gehlert et al. (2023) show that the impulse-based method can capture the added mass force given sufficiently resolved boundary layer data. However, such data are not available in this study due to limited visibility on the pressure side of the wing, as caused by the projection effect explained in Fig. 5.

Baddoo et al. (2020) propose a method to obtain exact solutions of potential flow for the ground effect using conformal mapping. However, a simple mapping function for the Tyrrell wing is not available. Therefore, the added mass is computed from a potential flow solution obtained by a panel method (Katz and Plotkin 2001).

To conduct this computation, we use an open-source code (*JavaFoil*), where the ground no-flux boundary condition is enforced using the method of images, rather than explicitly meshing the ground (Hepperle 2017).

All equations in this section are formulated in the Lagrangian coordinate system introduced in Fig. 3, with the origin located at the geometric center of the wing.

The relevant added mass force component for the downforce is in the *z*(3) direction; see Fig. 3. The added mass force in the $x_i$ direction is given by Brennen (2006):

2 $$\begin{aligned} F_i = -M_{ij}a_j \end{aligned}$$

where $M_{ij}$ denotes the added mass in the $x_i$ direction due to acceleration in the $x_j$ direction, with *i*,*j* = 1,2,3; see Fig. 3.

In the present experiment, the motion of the wing is only in the *x*(1) direction, while the downforce acts in the *z*(3) direction; see Sect. 2. Hence, the relevant added mass component is $M_{31}$, representing the added mass in the *z*(3) direction due to acceleration in the *x*(1) direction, i.e.

3 $$\begin{aligned} F_3 = -M_{31}a_1. \end{aligned}$$

In the experiments, the acceleration $a_{1}$ is given as an output of the robot motion.

**Fig. 6:**
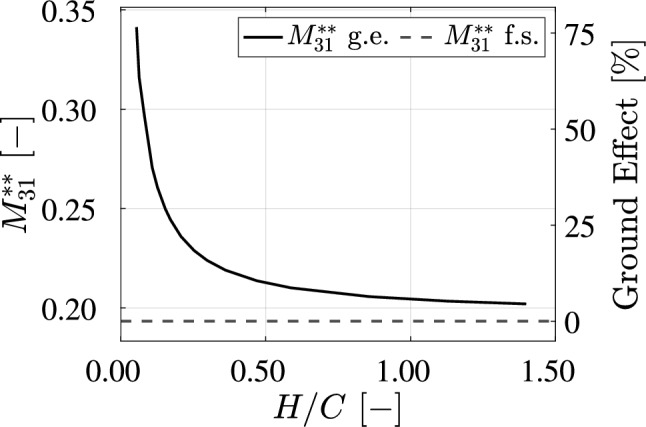
The added mass coefficient $$M^{**}_{31}$$, defined in Eq. 5, as a function of the normalized ground clearance *H*/*C*, where *C* is the wing chord. The added mass coefficient $$M^{**}_{31}$$ represents the non-dimensional added mass per unit width in the *z*(3) direction, as a result of acceleration in the *x*(1) direction. The right vertical axis indicates the relative strength of the ground effect, expressed as a percentage of the added mass coefficient under free-stream condition

The flow velocity $u_i$ that is the result of the object (i.e., wing) motion $U_j$ is written as: $u_i = u_{ij}U_j$, with: $u_{ij} = \partial \phi _j / \partial x_i$, where $\phi _j$ denotes the velocity potential of the steady flow due to motion with unit velocity in the *j* direction (Brennen 2006). By applying a panel method to find the flow potential $\phi$, the added mass $M_{31}$ can be computed with:

4 $$\begin{aligned} M_{31} = -\rho \oint _S \phi _3 \frac{\partial \phi _1}{\partial n} \textrm{d}S, \end{aligned}$$

where *S* is the wing surface, and *n* the outward surface normal (Brennen 2006). The added mass $M_{31}$ of the wing is computed for different ground clearance *H*, with the result shown in Fig. 6. The ground clearance is expressed in non-dimensional form as *H*/*C*, where *C* is the wing chord, and the added mass per unit width is expressed in non-dimensional form using the added mass coefficient (Brennen 2006):

5 $$\begin{aligned} M^{**}_{31} = \frac{M_{31}}{\rho B C^2}, \end{aligned}$$

where $\rho$ is the fluid density, and *B* the wing span.

As shown in Fig. 6, the non-dimensional added mass component $$M^{**}_{31}$$ drops significantly with increasing ground clearance. At the lowest values of the clearance considered here, the added mass $$M^{**}_{31}$$ is over 1.5 times the free-stream value. This highlights the strong influence of ground clearance on the contribution of the added mass force. This result of this calculation is used in the force reconstruction, discussed in subsubsect. 3.2.

#### Downforce measurement

**Fig. 7:**
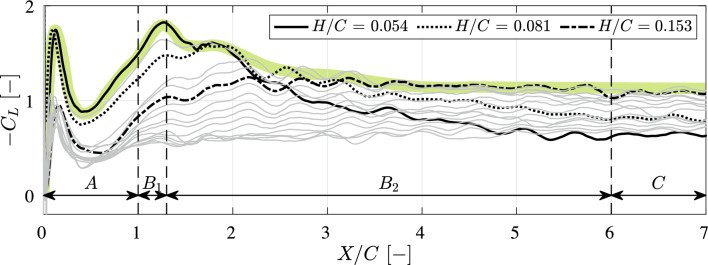
The measured lift coefficient $C_L$ (defined in Eq. 6) as a function of various normalized ground clearances *H*/*C* from $H/C = 0.054$ to $H/C = 1.40$. The results at $H/C = 0.054$ and $H/C = 0.153$ are highlighted in black, while the data for other *H*/*C* values exhibit similar trends. The motion is divided into three distinct phases: phase *A* corresponds to the acceleration phase; phases $B_1$ and $B_2$ represent the transition phase; and phase *C* denotes the steady phase. During phases *A* and $B_1$, the complete vorticity field is captured. During phase $B_1$, the initial trailing edge vortex (TEV) travels out of the cameras’ Field of View (FOV). Further details regarding to the vorticity field are provided in Fig. 9 and Fig. 10. The green band explained in section 4

The downforce *L* is measured at various ground clearances using the *F*/*T* transducer described in subsubsection 2.3. The results are shown in Fig. 7, where the lift coefficient $C_L$ is defined as:

6 $$\begin{aligned} C_L = \frac{2L}{ \rho \textrm{B} C U^2}, \end{aligned}$$

where *U* is a function of the non-dimensional traveled distance *X*/*C* introduced in Fig. 4.

Given that the downforce *L* is negative in our coordinate system, so that $C_L < 0$, we present the lift coefficient often as $-C_L$ in subsequent graphs.

As shown in Fig. 7, the motion is divided into three distinct phases based on the non-dimensional traveled distance *X*/*C*: the acceleration phase **A** ($0 \leqslant X/C < 1$); a transition phase **B** ($1\leqslant X/C <6$), and the stationary-motion phase **C** ($6\leqslant X/C \leqslant 7$). The transition phase is subdivided into phases $B_1$ and $B_2$, where only phase $B_1$ offers full visibility of the rotational contributions to the flow field. This definition of the phases is used in the remainder of the paper. In Fig. 7, the solid line represents the case with the highest $-C_L$ during the acceleration phase ($H/C = 0.054$), while the dashed line corresponds to the case with the highest $-C_L$ during the steady phase ($H/C = 0.153$).

At the start of the acceleration phase, a starting peak in $C_L$ is observed from $X/C = 0$ to $X/C = 0.5$. This is due to the added mass introduced by the sudden engagement of the robot. Following this peak, $-C_L$ increases until the end of the acceleration phase. Although the rate of increase (i.e., slope) is nearly identical in all cases, the starting values around $X/C=0.5$ differ. In general, the magnitude of $-C_L$ is inversely correlated with ground clearance *H*/*C*: when the wing operates at lower ground clearance, it generates a larger downforce and does so more rapidly.

In the $B_1$ phase (immediately following the acceleration phase), the value of $-C_L$ continues to increase, reaching a local peak around $X/C = 1.2$. This peak is referred to as the $B_1$-*peak*.

In the $B_2$ phase, the value of $-C_L$ decreases at low ground clearance. For $H/C < 0.081$, the rate of decrease increases as the ground clearance decreases. In contrast, for $H/C> 0.153$, the value of $-C_L$ continues increasing with a slower rate, until it remains approximately constant. For $H/C \ge 0.081$ and $H/C \le 0.153$, the value of $-C_L$ continues increasing with a slower rate, until it reaches a peak at around $X/C=2$. And then the value of $-C_L$ reduces slowly to a constant value. The perturbations observed in the force signal during this phase are attributed to vortex shedding. The relationship between the vorticity field and force behavior is further examined in subsect. 3.2.

In the steady phase *C*, the value of $-C_L$ stabilizes and remains constant for all ground clearances.

**Fig. 8:**
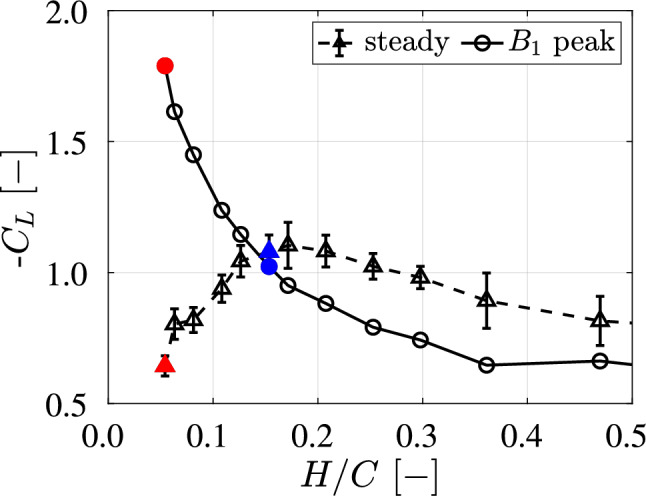
Measured lift coefficient $C_L$, defined in Eq. 6, evaluated at the $B_1$ peak (circles) and its time-averaged value in the steady phase *C* (triangles). The phases $B_1$ and *C* are defined in Fig. 7. The $B_1$ peak corresponds to the maximum value of $-C_L$ observed within phase $B_1$, while the steady phase $C_L$ represents the average of all measured values during phase *C*

We use the $B_1$-peak value of $-C_L$ to characterize the wing performance during acceleration; *vice versa*, the time-averaged value of $-C_L$ represents the wing performance during steady motion (phase *C*). As shown in Fig. 8, the case with the lowest ground clearance *H*/*C* generates the highest $-C_L$ during acceleration (red circle), but also gives the lowest value of $-C_L$ in the steady phase (red triangle). In contrast, the case at $H/C = 0.153$ achieves one of the highest values of $-C_L$ during steady motion (blue triangle), while its corresponding downforce during acceleration (blue circle) is approximately 80% lower than that of the lowest ground clearance case.

These results indicate that the optimal ground clearance to maximize downforce depends on the type of motion. For steady motion, a ground clearance around $H/C = 0.153$ appears to be optimal for this wing configuration. However, during acceleration, the optimal clearance changes to $H/C = 0.054$, or lower. To further investigate the differences in the generated downforce, PIV measurements are conducted on the two cases that show the best performance during acceleration ($H/C = 0.054$) and steady motion ($H/C = 0.153$), respectively.

#### Vorticity field

**Fig. 9:**
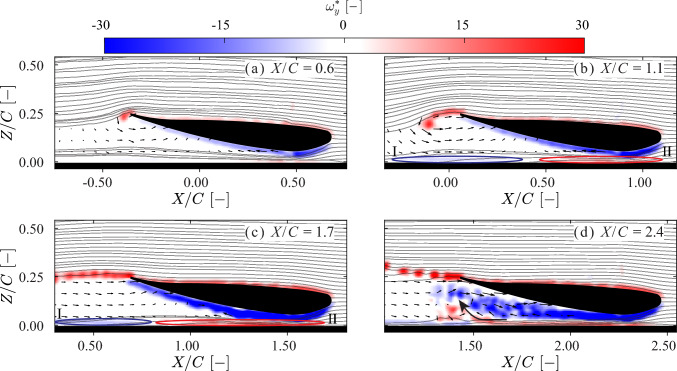
Snapshots of non-dimensional out-of-plane vorticity field $\omega ^*_y$ = $\omega _y C/U$, where *C* is the wing chord and *U* the wing velocity at normalized ground clearance $H/C = 0.054$ in the acceleration (*A*) and transition ($B_1$ &$B_2$) phases (defined in Fig. 7). Overlaid are streamlines that do not enter the wake region. Quivers are plotted every 10 data points in the wake region. The shape of the wing and the false bottom are represented in solid black. **a** In the acceleration phase (*A*), the formation of a trailing edge vortex (TEV) is observed. A Venturi effect is observed between the suction side of the wing and the false bottom. **b** In the early transition phase ($B_1$), the initial TEV is still within the field of view (FOV) and is advected forward (in the Eulerian reference frame) and downward. A TEV sheet follows, extending downward. Two opposing boundary layers, I and II, highlighted by blue and red ellipses, are observed on the false bottom. **c** & **d** In the later transition phase ($B_2$), part of the vorticity moves out of the FOV. **c** The suction-side boundary layer begins to dissipate. **d** The two opposing boundary layers on the false bottom begin to merge, generating an ’upwash’ (marked out by a curved black arrow). With influence from the upwash, the TEV sheet breaks into a series of vortices directed upward. The suction-side boundary layer continues to separate, and contributes to the formation of a wake region. This wake merges with the vortex group generated by the merging of the two opposing boundary layers on the false bottom. A corresponding movie is available as Supplementary Material

**Fig. 10:**
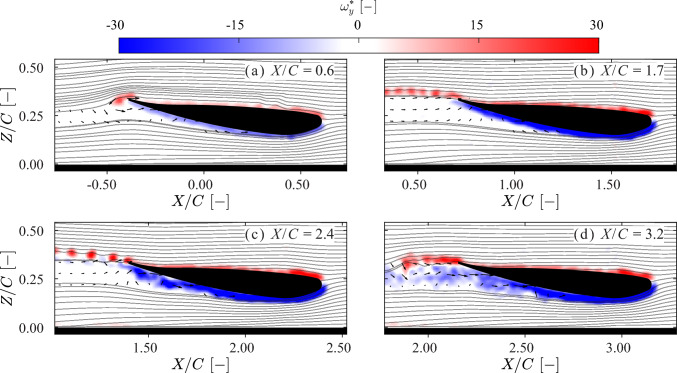
Snapshots of non-dimensional out-of-plane vorticity field $\omega ^*_y$ = $\omega _y C/U$, where *C* is the wing chord and *U* the wing velocity at normalized ground clearance $H/C = 0.153$ in the acceleration (*A*) and transition ($B_2$) phase (defined in Fig. 7). Quivers are plotted every 10 data points in the wake region. **a** In the acceleration phase (*A*), the formation of a trailing edge vortex (TEV) is observed. **b** In the transition phase ($B_2$), at $X/C=1.7$, the suction-side boundary layer remains attached and does not separate. It generates an upwash that advects the TEV sheet upward. **c** At $X/C=2.4$, the suction-side boundary layer intensifies and begins to separate. The resulting upwash breaks the TEV sheet into a series of vortices with positive $\omega ^*_y$, which are further advected upward. **d** As the suction side boundary layer continues to separate, a wake region forms. Due to the low-pressure zone in the wake region, the angle of the TEV sheet is reduced, although the TEV sheet still points upward. A corresponding movie is available as Supplementary Material

Figure 9 and Fig. 10 show the vorticity fields around the wing at two different ground clearances, as discussed in the previous section. These flow fields are captured using PIV (as described in subsect. 2.4) during the motion illustrated in Fig. 4. The out-of-plane component of the vorticity $\omega _y$ is presented in dimensionless form: $\omega _y^*$ = $\omega _yC/U$, where *C* is the wing chord, and *U* is the wing velocity.

During the acceleration phase, the boundary layers on both the pressure and the suction sides of the wing remain attached. For both clearance cases, the initial TEV exhibits a similar intensity and structure. A TEV sheet forms after the initial TEV and interacts with the boundary layer on the suction side, breaking into multiple shedding TEVs. These shedding vortices may account for the oscillations observed in the force signals shown in Fig. 7, as the shedding frequency and force oscillations are both approximately 5 Hz.

At $H/C = 0.054$, a stronger boundary layer is generated on the suction side at the lowest point of the wing. As the flow progresses toward the trailing edge, the intensity of the suction side boundary layer decreases. Two opposing boundary layers are observed on the false bottom, introducing an upwash flow near the ground after they merge. After the acceleration phase, the suction side boundary layer begins to separate; see Fig. 9 c & d. At $X/C = 2.4$, this separated boundary layer develops into a turbulent wake region. The two opposing ground boundary layers merge to form a positive vorticity region (shown in red) beneath the wake region. This enhances the upwash and reorients the TEV sheet to point upward.

At $H/C = 0.153$, the suction side boundary layer starts to separate later than in the case for $H/C = 0.054$; see Fig. 10c. No vortex–ground interaction is observed. An upwash flow is still present; however, unlike in the previous case, it is induced by the well-attached suction side boundary layer rather than the ground-bound vortices identified in Fig. 9d. This upwash also causes the TEV sheet to point upward. Due to the absence of merged ground boundary layers, the wake region is narrower than in the case for $H/C = 0.054$. After the wake forms, the upwash effect appears to diminish.

A comparison of the vorticity fields in the two cases reveals substantial differences. To better understand the role of vorticity in the generation of downforce, the downforce is reconstructed from the measured vorticity fields using the methodology of Gehlert et al. (2023); the results of of this are discussed in the next section.

### Downforce reconstruction

#### Methods

We used the impulse-based method introduced by Gehlert et al. (2023) to reconstruct the downforce. For comparison, we also apply the Kutta-Joukowski lift theorem (Kundu et al. 2015).

The impulse-based method reconstructs the downforce according to Gehlert et al. (2023):

7 $$\begin{aligned} -L = -\rho \int _{R_{wing}}\left( \underbrace{\frac{\textrm{d}I_z^{wing}}{\textrm{d}t}}_{\textrm{impulse}}+\underbrace{U_{\omega ^{wing}}\omega ^{ext}}_{\textrm{advection}} \right) \textrm{d}R, \end{aligned}$$

which combines two primary effects: the impulse effect introduced by the vorticity generated by the wing, and the advection effect introduced by the vorticity generated from external bodies, such as the ground. In Eq. 7 the impulse $I_z^{wing}$ is given by:

8 $$\begin{aligned} I_z^{wing} = x\omega ^{wing}, \end{aligned}$$

with $\omega ^{wing}$ denoting the vorticity generated by the wing.

In the advection term, $U_{\Omega ^{wing}}$ is computed based on the Biot-Savart law (Gehlert et al. 2023):

9 $$\begin{aligned} U_{\omega ^{wing}} = \frac{\int _{R_{wing}}\omega ^{wing}\textrm{d}R}{2\pi r}, \end{aligned}$$

where *r* is the distance vector from the center of $\omega ^{wing}$ to a point of interest outside of $R_{wing}$.

To apply Eq. 7 in practice, the flow field is partitioned into two distinct regions, each comprising different flow structures (see Fig. 1). The region $R_{wing}$ encompasses all vorticity generated by the wing ($\omega ^{wing}$), including the wake region, the TEV sheet, and the boundary layer of the wing; the remaining region contains only the external vorticity $\omega ^{ext}$, which is generated by the ground, marked as $\omega ^{ext}_{ground}$. A more detailed illustration of the application of the impulse-based method is provided in the Appendix.

For comparison, we also consider the downforce computed with the circulation-based Kutta-Joukowski lift theorem

10 $$\begin{aligned} L = \rho U \Gamma , \end{aligned}$$

with the circulation $\Gamma$ is given by

11 $$\begin{aligned} \Gamma = \oint _{c} {\textbf {u}}\cdot \textrm{d} {\textbf {l}}, \end{aligned}$$

where *c* is a contour enclosing the wing.

#### Downforce reconstruction

**Fig. 11:**
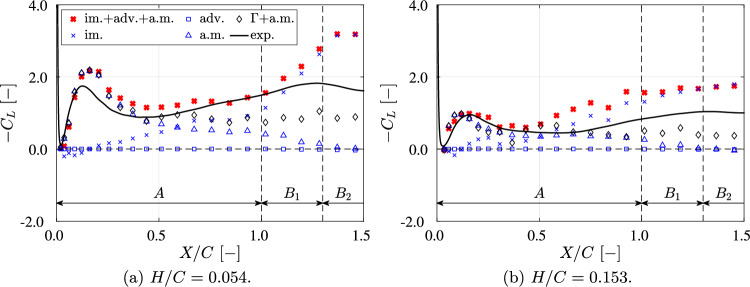
The normalized reconstructed downforce, denoted as $-C_L$, is plotted against the normalized traveled distance *X*/*C*. The reconstruction of downforce using the impulse-based method (red crosses) is obtained by summing the impulse force (im.), the advection force (adv.), and the added mass force (a.m.); the circulation-based method reconstructs downforce (diamonds) by summing the circulation-based force ($\Gamma$) and the added mass force (a.m.). In the acceleration phase, the downforce reconstructed by the impulse-based method mostly agrees with the data measured by the force/torque transducer (black solid line). However, in the transition phase ($B_1$), the reconstructed force begins to deviate due to missing data in the vorticity field. **a** At $H/C=0.054$, the added mass force (blue triangles) is dominant at $X/C<0.5$, while the impulse force (blue crosses) becomes dominant at $X/C>0.5$. **b** At $H/C=0.153$, the added mass force is dominant at $X/C<0.5$, with the impulse force taking over at $X/C>0.5$

**Fig. 12:**
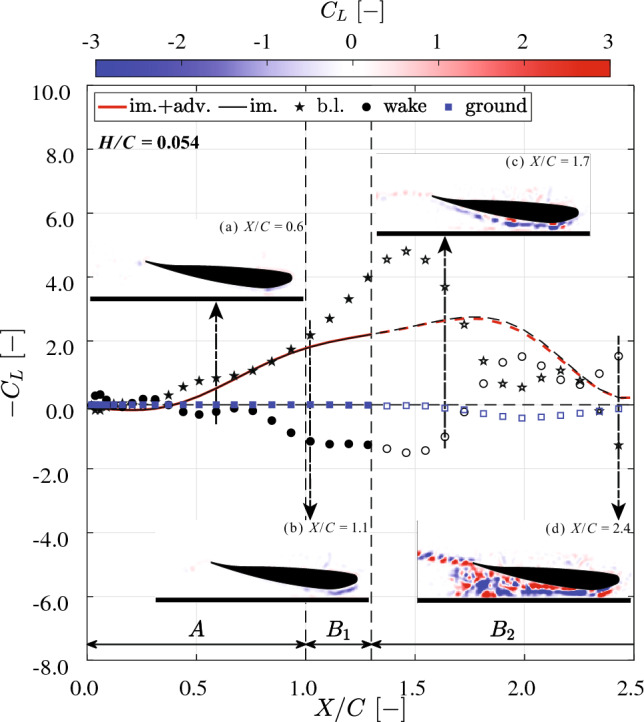
Contributions of different flow regions to the normalized downforce ($-C_L$, as defined in Equation.6), reconstructed using the impulse-based method during the acceleration (*A*) and transition ($B_1$ &$B_2$) phases, at a normalized ground clearance $H/C = 0.054$. Circular markers indicate contributions from vorticity in the wake region, including the turbulent wake, trailing edge vortex (TEV), and TEV sheet (defined in Fig. 1). Due to the vorticity moving out of the field of view (FOV) (Fig. 9) after the $B_1$ phase, the downforce reconstruction results in phase $B_2$ are shown as open markers. The inset shows four snapshots of the lift coefficient $C_L$ contour, where regions contributing positively to the downforce are shown in blue. **a** Downforce is primarily generated by the suction-side boundary layer. The TEV also contributes positively, but its effect is largely offset by the negative contribution from the trailing TEV sheet. **b** In the early transition phase ($B_1$), the boundary layer remains the dominant contribution to downforce, while the TEV sheet continues to have a negative effect. **c** In the later transition phase ($B_2$), at $X/C = 1.7$, as boundary layer dissipation begins (see Fig. 9), its contribution to downforce is reduced. Meanwhile, the boundary layer on the ground begins to contribute negatively. **d** At $X/C = 2.4$, the turbulent wake becomes the dominant source of downforce. Following the detachment of the suction-side boundary layer (see Fig. 9), the boundary layer region exhibits a negative contribution to downforce

**Fig. 13:**
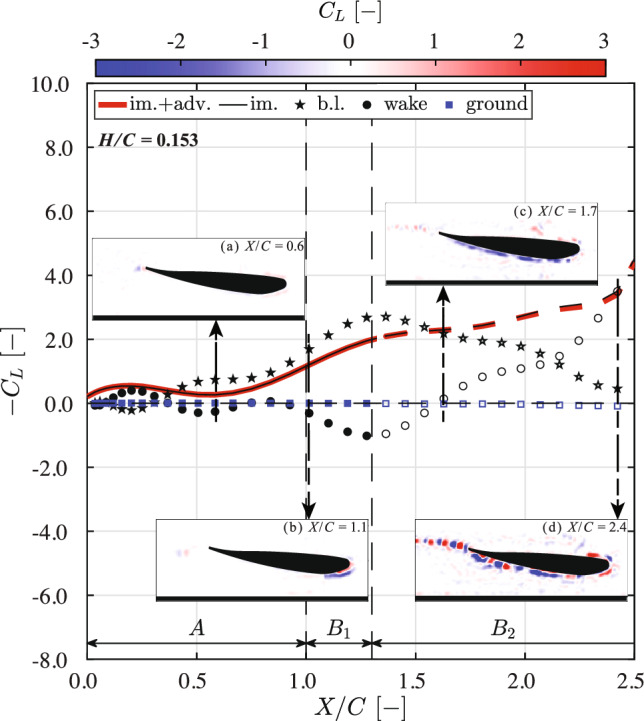
Contributions of different flow regions to the normalized downforce ($-C_L$, as defined in Eq. 6), reconstructed using the impulse-based method during the acceleration (*A*) and transition ($B_1$ &$B_2$) phases, at a normalized ground clearance $H/C = 0.153$. Circular markers indicate contributions from vorticity in the wake region, including the turbulent wake, trailing edge vortex (TEV), and TEV sheet (defined in Fig. 1). Due to the vorticity moving out of the field of view (FOV) (Fig. 10) after the $B_1$ phase, the downforce reconstruction results in phase $B_2$ are shown as open markers. The inset shows four snapshots of the lift coefficient $C_L$ contour, where regions contributing positively to the downforce are shown in blue. **a** & **b** In the acceleration (*A*) and early transition ($B_1$) phases, similar downforce reconstruction patterns are observed compared to the lower ground clearance case as shown in Fig. 12. The contribution from the boundary layer is reduced at this higher clearance. **c** In the later transition phase ($B_2$), at $X/C=1.7$, the TEV sheet contributes positively to the downforce due to the stronger ’upwash’ induced by the non-dissipating suction-side boundary (as introduced in Fig. 10). **d** At $X/C=2.4$, the suction-side boundary layer is dissipated, and the wake region dominates the downforce generation, while the boundary layer’s contribution is diminished

The downforce is reconstructed using the two methods described in the previous subsection. Figure 11 shows the reconstruction results based on the terms in Eq. 7, while Figs. 12 and 13 show the reconstruction results based on specific flow structures; this is further discussed in subsubsect. 3.2.3. The results of the downforce *L* for both reconstructions are presented in non-dimensional form as $-C_L$ using Eq. 6.

As shown in Fig. 11, before phase $B_2$, the generally reconstructed downforce (red filled crosses) agrees well with the measurements obtained from the force/torque (F/T) transducer. The mismatch observed in phase $B_2$ is attributed to the fact that some vortices move out of the FOV and three-dimensional flow structure. Consequently, the reconstructed values for $-C_L$ are overestimated as a consequence of vortices moving out of the FOV and the three-dimensional flow. This is further discussed in subsubsect. 3.2.3.

At $H/C = 0.054$, the added mass contribution (blue triangles) dominates until $X/C = 0.5$. The impulse term (blue crosses) begins to increase after $X/C = 0.5$ and becomes dominant beyond $X/C = 0.7$. The contribution of the advection term (blue rectangles) remains negligible for $X/C < 1.5$.

At $H/C = 0.153$, the added mass and impulse terms contribute equally to the downforce until $X/C = 0.5$. Beyond this point, the impulse term becomes dominant. The contribution of the added mass force is much smaller compared to the case for $H/C = 0.054$; this is the result of the drop in added mass for larger ground clearance, as shown in Fig. 6). The impulse term shows a similar trend compared to the case for $H/C = 0.054$ before $X/C = 0.4$; however, beyond this point, its rate of increase is decreases. The advection term, as in the previous case, remains negligible for $X/C < 1.5$.

The downforce reconstructed using the Kutta-Joukowski lift theorem in Eq. 10 (diamonds) clearly underestimates the actual downforce for both cases. This is expected due to the observed flow separation on the suction side of the wing, which significantly reduces the circulation $\Gamma$ and thus leads to a reduction of the reconstructed downforce.

#### Contribution of flow structures

To further investigate how different flow structures contribute to the terms in Fig. 11, the reconstructed downforce from the regions defined in Fig. 1 at $H/C=0.054$ and $H/C=0.153$ is presented in Figs. 12 and 13, respectively. The total reconstructed downforce (red lines) is composed of two parts: (i) the contribution from the wing-generated flow (i.e., the impulse term represented by the black lines), and: (ii) the contribution from the ground-generated flow (i.e., the advection term, represented by the blue rectangles). In addition to the force contributions of different flow structures, four representative snapshots of the lift coefficient contours are also shown at typical moments for each case, providing further insight into the aerodynamic behavior.

Due to the incomplete capturing of the full vorticity field in phase $B_2$ (see subsubsect. 3.2.2), the force reconstruction results in this phase are represented by open markers.

At $X/C<1.5$, in both cases, the downforce is primarily generated by the boundary layer on the wing (indicated by black stars). As *X*/*C* increases beyond 1.5, the contribution from the boundary layer begins to decrease. This is due to separation of the suction-side boundary layer and the development of the wake region. This transition is more prominent for the lower ground clearance of $H/C=0.054$ (see Fig. 9), where a greater change is observed in the thickness of the suction-side boundary layer and in the size of the wake region. The wake region can be identified more or less as the area that is void of streamlines originating from upstream of the leading edge of the wing. Since the turbulent wake vorticity is not yet established at $X/C < 1.5$, the only other contribution to the impulse term is the TEV sheet (black circles). The initial TEV provides a small but positive contribution to the downforce for $X/C<0.4$. However, for $X/C>0.4$, its contribution becomes negative due to the formation of a TEV sheet that is trailing the TEV. This can be confirmed by the lift coefficient contours in insets (a&b) of Fig. 12 and insets (a&b) in Fig. 13, where the initial TEV contributes positively to the downforce (blue contours), while the TEV sheet contributes negatively (red contours).

For $X/C>1.5$, the boundary layer separates and a turbulent wake forms, indicates as the ‘wake’ term in both Figs. 12 and 13, for which the contribution to the downforce increases and eventually surpasses that of the boundary layer. For $H/C = 0.054$, after the detachment of the suction-side boundary layer (see Fig. 9), the boundary layer region exhibits a negative contribution to the downforce. At $X/C = 2.4$, the lift coefficient contour reveals that the shedding TEVs produce an alternating pattern of positive and negative contributions to the downforce; see insets (d) in Figs. 12 and 13. This is similar to the contribution of the vorticity in the turbulent wake region beneath the suction side of the wing. In both cases, the ground-generated vorticity has negligible effect on the downforce during both the acceleration (*A*) and transition ($B_1$) phases. However, for $H/C = 0.054$, the contribution to the downforce in phase $B_2$ is negative, which appears to be the result of the formation and merging of the two opposing boundary layers at the ground, as shown in Fig. 9. In contrast, for $H/C = 0.153$, the contribution of the ground-generated vorticity remains negligible, as no significant vorticity is generated near the ground; see also Fig. 10.

Comparing Figs. 7, 12, and 13, it is evident that the reduction in downforce observed at $H/C = 0.054$ after phase $B_1$ is the result of the absence of downforce generation by the wake region. In contrast,, the continued increase in downforce for $H/C = 0.153$ after phase $B_1$ is primarily due to an increase in the contributions from the wake region. The difference in wake-generated downforce arises from the influence of ground-generated vorticity at the lower clearance of $H/C = 0.054$. In practical applications, such vortex interactions should be avoided, as they appear to diminish the aerodynamic performance of the wing in ground effect.

## Discussion and conclusion

In this research, the downforce and flow field of a model F1 front wing in ground effect during both accelerating and steady motions are investigated. The downforce of the wing is measured using a force/torque (F/T) transducer, while the flow field is captured using planar particle image velocimetry (PIV) with two cameras that move along with the wing.

From the force measurements, the maximum steady motion downforce is measured at a normalized ground clearance of $H/C = 0.153$. For a lower ground clearance a higher peak in the downforce was measured at the end of the acceleration phase.

The downforce is also reconstructed from the flow field measured by PIV, incorporating an added mass force prediction. As shown in Fig. 11, the added mass force significantly contributes to the downforce at $X/C < 0.5$, explaining the starting peak observed in the measured downforce in Fig. 7. As the measurements are done in water, the contribution of added mass to the downforce are significant, while these can be largely ignored for air flow, as is the case in F1 racing.

During the acceleration phase, the reconstructed downforce closely matches the measured downforce. Neglecting the added mass effect, the boundary layer is identified as the primary contributor to the downforce, while other flow regions contribute to a lesser extent. After the acceleration phase, the interaction between the wake region and the ground-generated vortical flow introduces a negative contribution to the downforce, although the wake itself continues to contribute positively to the downforce.

However, as soon as the trailing vortices start to exit the field of view (FOV), a mismatch emerges in the impulse-based force reconstruction. Although the out-of-FOV correction method, introduced by Corkery et al. (2019), can help reduce this mismatch, a significant discrepancy remains. This is likely due to the breakdown of the ‘infinitely far vortex’ assumption. In the present case, the FOV can capture vortices that are at most 4 cm from the wing. This distance is too close to satisfy the ‘infinitely far vortex’ assumption.

One of the results of this investigation is that a front wing with initially lower ground clearance may produce more downforce during acceleration. To maximize the overall downforce, strategies such as delaying boundary layer separation and suppressing early wake formation can also be effective. This could be achieved by gradually increasing the ground clearance during acceleration.

The green band in Fig. 7 indicates the maximum downforce during the entire motion, with initially a low ground clearance (*H*/*C* = 0.054) at the initiation of the motion and gradual increase of the ground clearance toward that in the steady-motion case (*H*/*C*=0.153). In addition, this may also help to avoid unwanted vortex-ground interactions, such as those shown in Fig. 9.

For future research, it is essential to extend the field of view (FOV) of the PIV to ensure that no vortical structures leave the measurement domain. It is also important to resolve the pressure side boundary layer for a more complete analysis. Beyond acceleration, the effects of deceleration and cornering are both critical dynamic motions in F1 racing. These topics remain largely unexplored in the context of the ground effect. Furthermore, the drag force is another key aerodynamic component that requires further detailed investigation. A comprehensive understanding of these dynamic motions and their aerodynamic impacts could help improve lap times and provide a competitive advantage in F1 racing.

## Supplementary Information

Below is the link to the electronic supplementary material.

Supplementary file 1 (mp4 10884 KB)

Supplementary file 2 (mp4 8713 KB)

## Data Availability

The data are made available upon request.

## Appendix

### Application of impulse-based method

To apply the impulse-based method, a reformulation of Eq. 7 is preferred. We begin by splitting it into two distinct components:

12 $$\begin{aligned} \begin{aligned} L&= \underbrace{\rho \int _{R_{wing}}\frac{\textrm{d}I_z^{wing}}{\textrm{d}t}\textrm{d}R}_{\textrm{impulse}}\\&+\underbrace{\rho \int _{R_{wing}}U_{{\omega ^{wing}}_i}\omega ^{ext}_j\textrm{d}R}_{\textrm{advection}}, \end{aligned} \end{aligned}$$

where $U_{{\omega ^{wing}}_i}$ denotes the velocity induced by the *i*-component of the vorticity in the region $R_{wing}$, as given by the Biot-Savart law, and $\omega ^{ext}_j$ is the *j*-component of the vorticity located in the external region. For the impulse term, the order of integration and differentiation can be exchanged, based on the Reynolds transport theorem (RTT) introduced by Kundu et al. (2015). The advection term is rewritten as:

13 $$\begin{aligned} \begin{aligned} \int _{R_{wing}}U_{{\omega ^{wing}}_i}\omega ^{ext}_j\textrm{d}R =\\ \int _{R_{wing}}U_{\omega ^{wing,p}_{ext}}\sum _{i=1}^{N}\omega ^{ext,p}_{i}\textrm{d}R+\\ \int _{R_{wing}}U_{\omega ^{wing,n}_{ext}}\sum _{i=1}^{N}\omega ^{ext,p}_{i}\textrm{d}R+\\ \int _{R_{wing}}U_{\omega ^{wing,p}_{ext}}\sum _{i=1}^{N}\omega ^{ext,n}_{i}\textrm{d}R+\\ \int _{R_{wing}}U_{\omega ^{wing,n}_{ext}}\sum _{i=1}^{N}\omega ^{ext,n}_{i}\textrm{d}R, \end{aligned} \end{aligned}$$

where $U_{\omega ^{wing,p}_{ext}}$ is the velocity induced by the vorticity with positive sign in the region $R_{wing}$, as given by the Biot-Savart law, evaluated at the weighted average location of the external vorticity with positive sign *p* or negative sign *n*. Substitution of Eq. 13 in Eq. 12 yields:

14 $$\begin{aligned} \begin{aligned} L&= \underbrace{\rho \frac{\textrm{d}\left( \int _{R_{wing}}I_z^{wing}\textrm{d}R\right) }{\textrm{d}t}}_{\textrm{impulse}}\\&+\underbrace{\rho \int _{R_{wing}}U_{\omega ^{wing,p/n}_{ext}}\sum _{i=1}^{N}\omega ^{ext,p/n}_{i}\textrm{d}R}_{\textrm{advection}}. \end{aligned} \end{aligned}$$

Next, we introduce the discrete forms of Eqs. 8 and 9 in Eq. 14:

15 $$\begin{aligned} \begin{aligned} L&= \underbrace{\rho \frac{\sum _{j=1}^{M}I_{{z}^{wing}_j}\textrm{d}x_{j}\textrm{d}y_{j}}{\textrm{d}t}}_{\textrm{impulse}}\\&+\underbrace{\rho \frac{\sum _{j=1}^{M}\omega ^{wing,p/n}_{j}\textrm{d}x\textrm{d}y}{2\pi \bar{r}}\sum _{i=1}^{N}\omega ^{ext,p/n}_{i}\textrm{d}x\textrm{d}y}_{\textrm{advection}}, \end{aligned} \end{aligned}$$

where $\bar{r}$ is the vector from the weighted average location of the internal vorticity ($\omega ^{wing}$) to that of the external vorticity, and $\textrm{d}x\textrm{d}y$ represents the physical area associated with a single interrogation window in the flow domain. This implementation of the impulse-based method improves the computational efficiency; it avoids the computationally intensive matrix operations present in the original expression in Eq. 7.

## Footnotes

1 www.the-black-market.com/marketplace/musou-black-paint

## References

[CR1] Adrian RJ, Westerweel J (2011) Particle Image Velocimetry, Cambridge Aerospace Series, vol 30. Cambridge University Press, Cambridge, UK

[CR2] Arrondeau B, Rana ZA (2020) Computational aerodynamics analysis of non-symmetric multi-element wing in ground effect with humpback whale flipper tubercles. Fluids 5(4):247

[CR3] Baddoo PJ, Kurt M, Ayton LJ et al (2020) Exact solutions for ground effect. J Fluid Mech 891:R2

[CR4] Brennen CE (2006) An Internet Book on Fluid Dynamics. http://brennen.caltech.edu/fluidbook/

[CR5] Corkery S, Babinsky H, Graham W (2019) Quantification of added-mass effects using particle image velocimetry data for a translating and rotating flat plate. J Fluid Mech 870:492–518

[CR6] Cui E, Zhang X (2010) Encyclopedia of Aerospace Engineering, John Wiley & Sons, chap 18 Ground Effect Aerodynamics

[CR7] Doig G, Barber T (2011) Considerations for numerical modeling of inverted wings in ground effect. AIAA J 49:2330–2333. 10.2514/1.J051273

[CR8] Gasparetto T, Orlova M, Vernikovskiy A (2024) Same, same but different: analyzing uncertainty of outcome in formula one races. Managing Sport and Leisure 29:651–665

[CR9] Gehlert P, Andreu-Angulo I, Babinsky H (2023) Vortex force decomposition-forces associated with individual elements of a vorticity field. Exp Fluids 64:112

[CR10] Grift E, Vijayaragavan N, Tummers M et al (2019) Drag force on an accelerating submerged plate. J Fluid Mech 866:369–398

[CR11] He Y, Qu Q (2014) Agarwal RK (2014) Shape optimization of an airfoil in ground effect for application to wig craft. J Aerodynam 1:931232

[CR12] Hepperle M (2017) Wing in ground proximity (wig). https://mh-aerotools.de/airfoils/jf_wig.htm

[CR13] Katz J (2006) Aerodynamics of race cars. Annu Rev Fluid Mech 38:27–63

[CR14] Katz J, Plotkin A (2001) Low-speed aerodynamics, vol 13. Cambridge University Press

[CR15] Knapik J, Gallyamov R, Ovchinnikov V, et al (2018) F1 car-front wing CFD analysis and optimization. In: Information Technology and Nanotechnology, pp 1875–1884

[CR16] Kundu PK, Cohen IM, Dowling DR (2015) Fluid Mechanics. Academic press, United States

[CR17] Lee T, Lin G (2022) Review of experimental investigations of wings in ground effect at low Reynolds numbers. Front Aerosp Eng 1:975158

[CR18] Pistolesi E (1937) Ground effect – theory and practice. Tech Rep. TM-828, NACA

[CR19] Preosti E (2021) Data analysis for Formula 1. Berkeley Sci J 25(2):53–56

[CR20] Quinn DB, Moored KW, Dewey PA et al (2014) Unsteady propulsion near a solid boundary. J Fluid Mech 742:152–170

[CR21] Toet W (2013) Aerodynamics and aerodynamic research in Formula 1. Aeronautical J 117:3929

[CR22] Zerihan J (2001) An investigation into the aerodynamics of wings in ground effect. PhD thesis, University of Southampton

[CR23] Zhang X, Toet W, Zerihan J (2006) Ground effect aerodynamics. Appl Mech Rev 59:33–49

